# Diphtheria and Its Cause

**Published:** 1882-03

**Authors:** 


### DIPHTHERIA AND ITS CAUSE.

SCIENTIFIC men tell of organisms so small that the highest powers of the microscope are requisite to determine anything about them, and whose powers of multiplication are so rapid that they would fill the earth in a brief time if not checked by natural means.

Whether these mites came from previously existing mites or from dead matter is not important. These bacteria, for such we call them, force themselves upon our notice—because it is asserted that they are the active agents in producing among men such diseases as measles, whooping cough, scarlet fever, diphtheria and the like; in lower animals producing charbon, chicken cholera, sheep rot and other similar diseases. These organisms are of vegetable nature and approach more nearly to the fungi than to any other group ; and will probably prove to be specific in character; that is, each kind produces a special disease.

Prof. H. C. Wood gave a history of them and of their relations to diphtheria.

He inoculated rabbits with diphtheritic membrane. These animals died, but few if any of them with diphtheria ; most succumbed to lung disease. This consumption, however, was proved not to be a direct result of the diphtheritic poison. So that experiment settles no main issue.

Next he inserted the diphtheritic membrane in the opened windpipe of the animals. This produced sore throat and membrane, which was nearly like that of diphtheria. Here, however, were found abundance of globular, transparent bodies, which when magnified several thousand times are no larger than pin-heads. These bodies are bacteria specially known as micrococci. Further it was shown that these micrococci may also be present in ordinary sore throat.

Last spring a fearful epidemic of diphtheria prevailed in Ludington, Mich., which is said to have destroyed about one-third of the children attacked in the place, and most of them were so taken. From material obtained there it was found that the blood of these diphtheritic patients were full of micrococci, often forming masses when the individuals were compacted by growth. They were found in the white blood corpuscles and also discovered blocking up and distending the blood vessels of the kidney. The more there are of these in the blood evidently the worse is the attack.

Inoculating animals (rabbits) with the diphtheric poison from Ludington, it resulted in a genuine attack of diphtheria, which proved fatal in a few days. The disease was simply that of Ludington, except that it was produced in the lower animals. Post mortem examination of the rabbits showed that even the bone marrow was full of micrococci. So with material obtained from the diphtheritic rabbits he inoculated others, and so through several sets of rabbits, producing death in each instance from diphtheria. Further experiments proved that these micrococci were either the direct cause of diphtheria or that they carried the poison which was. In either alternative, we can look upon them as offending parties.

Now, as in ordinary sore throat, in mild diphtheria, and in the malignant form, these micrococci show no constant differences of structure under high powers of the microscope, to what is the different grade of the disease they produce due? Simply to the reproductive capacity of the micrococci. Thus, those taken from mild forms of diphtheria would only reproduce up to the fifth generation; these from Ludington speedily ran through ten generations.

Experiments have shown that there are certain conditions under which these organisms lose their reproductive capacity to a great degree, and hence with it their power for harm. Hence the great problems in the healing art are: What will destroy this out of the body and so prevent the disease from arising; or what will kill them or lessen this power of reproduction in the body when the disease is contracted, and at the same time not injure the patient?

Signs of great hope may appear in this connection. It would hardly be putting the case too strongly to assert, there is a fair probability that by a process of modified inoculation, comparable to that against small-pox, medical men will be able to hold this and other like diseases in check, and that this triumph may be witnessed before the century goes out. If so, it will be the foundation on which the future historian will erect the noblest monument to our times.

